# Argon–Helium Cryoablation for Cutaneous Squamous Cell Carcinoma in the Elderly

**DOI:** 10.3389/fonc.2021.788490

**Published:** 2022-01-14

**Authors:** Qianwen Huang, Wenshen Xu

**Affiliations:** Department of Medical Oncology, Boluo County People’s Hospital, Huizhou, China

**Keywords:** argon, helium, cryoablation, cutaneous squamous cell carcinoma, therapeutic strategy

## Abstract

Cutaneous squamous cell carcinoma (cSCC) is a common type of malignant neoplasm in non-melanoma skin cancer (NMSC). Most cases of simple cSCC are considered curable by surgical removal of the lesion. However, clinical treatments for cSCC with medium- or large-sized lesions are difficult. Meanwhile, the effectiveness of the treatments is not guaranteed, especially for elderly patients, because of an intolerance to surgical resection or other adjuvant modalities. In such cases, safe and effective treatments with excellent aesthetic outcomes are urgently needed. In this study, we reported 6 elderly cSCC patients with medium- or large-sized lesions treated with argon–helium cryoablation. The average age of all 6 patients was 78 years (range 72–85 years). They were all diagnosed with cSCC with a median tumor size of 5.8 cm (range 2.5–15.5 cm) and dermal invasion. Complete ablation was achieved in all cases after a single ablation session (2 freeze–thaw cycles). Patients experienced mild pain and hemorrhage after ablation, but the symptoms were manageable. One patient developed infection and fever because of extensive necrosis of the tumor, which was eventually cured after treatment. All patients obtained good cosmetic outcomes, and their quality of life improved significantly. In the 5-year follow-up study, 4 patients were alive while 2 patients died of unrelated diseases 3 years after cryotherapy. None of the 6 patients had a recurrence. These results suggested the feasibility of argon–helium cryoablation as a novel therapeutic strategy for elderly cSCC with medium- or large-sized lesions.

## Introduction

Cutaneous squamous cell carcinoma (cSCC) is the second most common non-melanoma skin cancer (NMSC) after basal cell carcinoma (BCC) (1). Clinically, cSCC usually occurs on the skin of the scalp, face, neck, extremities, and other locations with chronic sun exposure. Appearances of cSCC usually include scaly plaques, nodules, and verrucous lesions with ulcers, necrosis, and crusts found in the center (2, 3). Most cSCC can be cured by surgery. Compared with BCC, cSCC is more invasive, with a higher probability of local expansion and/or metastasis. Upon tumor metastasis, the prognosis of cSCC deteriorates, leading to increased mortality (4). In the past two decades, the incidence of cSCC has risen dramatically by 133% (5). As most cSCCs occur in patients over 60 years old, the incidence of cSCC is potentially further increased due to the prolongation of the life span and the improvement of screening protocols (6).

The standard treatment procedure for cSCC is the surgical resection of the tumor and its surrounding tissue using a variety of surgical procedures, including standard excision, Mohs microsurgery, curettage, and liquid nitrogen cryotherapy. Surgical procedures are usually followed by radiotherapy, chemotherapy, or targeted therapy for patients with local recurrence or metastases (2, 7–10). However, some patients may not be ideal candidates for the aforementioned treatments due to large tumor size and deep invasion, especially for the elderly or patients with poor physical conditions who are not suitable for surgery or other methods such as radiotherapy and systemic therapy.

Liquid nitrogen is usually used as a freezing medium in cryotherapy for the treatment of cSCC (11). In previous clinical treatments, cSCC with tumor size larger than 2 cm, deep invasion, and high-risk features is excluded from cryotherapy (12). However, the application of cryoablation in cSCC treatment using argon and helium as a freezing medium is lacking. In this study, we aimed to evaluate the safety and clinical efficacy of argon–helium cryoablation for medium- and large-sized cSCC in the elderly.

## Materials and Methods

### Patients

From January 2014 to December 2016, 6 elderly patients diagnosed with cSCC by histological examination were recruited for this study. The average age of all 6 patients was 78 years (range 72–85 years). The tumor diameter of 6 patients was between 2.5 and 15.5 cm (average size 5.8 cm). All patients were fully informed of the risks and benefits of the cryoablation procedure. Informed consent was signed by all patients.

### Argon–Helium Cryoablation Procedure

**Figure 1:**
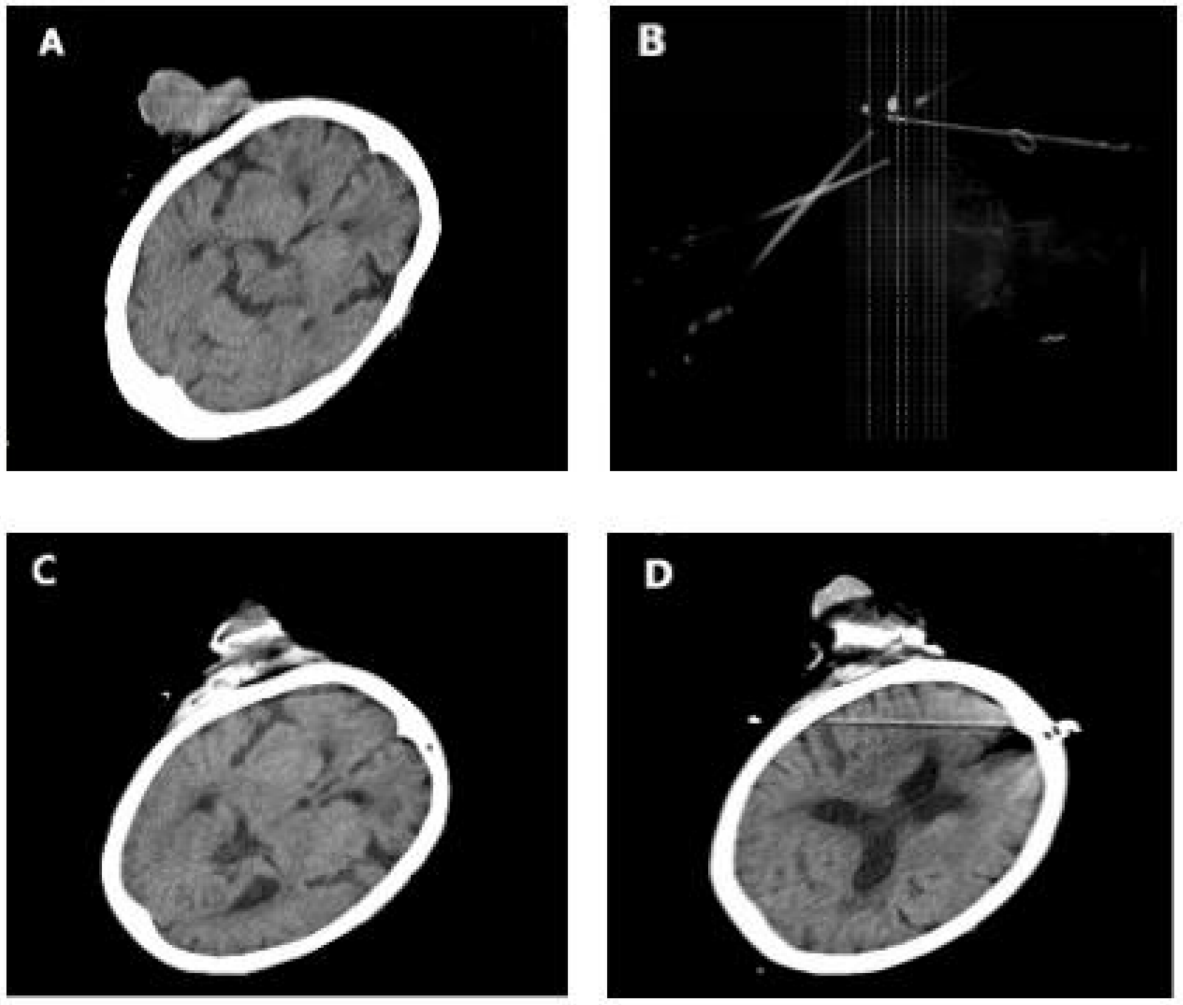
**(A)** Case 3 before cryotherapy. **(B)** Three cryoprobes were placed under CT guidance according to the size and location of the tumor. **(C, D)** During cryotherapy. The patients passed away due to unrelated diseases 3 years after cryotherapy.

Cryoablation was conducted using the cryoablation system (Cryo-hit) by an experienced interventional surgeon and was performed in a room equipped with a CT scanner. Preparation of the cryoablation procedure included position fixation, routine disinfection, and 2% lidocaine anesthesia. Under the guidance of CT imaging, a cryoprobe was inserted into the tumor (**Figure 1**). The number and position of cryoprobes used during the procedure were dependent on the size, shape, and location of the tumor. Each cryoprobe was 2.4 mm in diameter. Single or more cryoprobes could be operated at the same time. In general, an ice ball with a diameter of 3–4 cm was generated by one cryoprobe. Once the cryoprobe was satisfactorily placed, the freeze–thaw cycle was started (10–15 min freeze, 3–5 min thaw). Freezing was conducted using argon gas at a temperature of approximately −140°C, and thawing was conducted using helium gas. The freezing and thawing cycle was repeated once. During the cryoablation period, the sizes of ice balls were monitored *via* CT scans in real time. When necessary, additional cryoprobes were placed to attain full coverage of the lesion. Vital signs and reactions of all patients were closely observed for 24 h after the procedure.

### Postoperative Follow-Up

After the surgery, all patients were observed for 24 h and discharged from the hospital if no complication was found. All patients had mandatory return visits 2 and 4 weeks after the surgery. Upon complete ablation of the tumor, follow-up was conducted after 6 and 12 months and yearly thereafter. The total follow-up period lasted for 5 years.

## Results

A total number of 6 patients were treated with CT-guided argon–helium cryoablation. The clinical characteristics and distribution of the lesions in 6 patients with cSCC are displayed in **Table 1**. The average age of the 6 patients at the time of diagnosis was 78 years (range: 72 to 85 years). The maximum tumor diameter ranged from 2.5 to 15.5 cm, with a median diameter of 5.8 cm.

**Table 1:** Patient characteristics and distribution of the cSCC lesion.

Case no.	Age (year)	Sex	Location	Maximum tumor diameter (cm)
1	80	Female	Face	15.5
2	72	Female	Face	5
3	76	Female	Face	6
4	79	Female	Face	3
5	76	Male	Leg	2.5
6	85	Male	Leg	3

cSCC, cutaneous squamous cell carcinoma.

**Figure 2:**
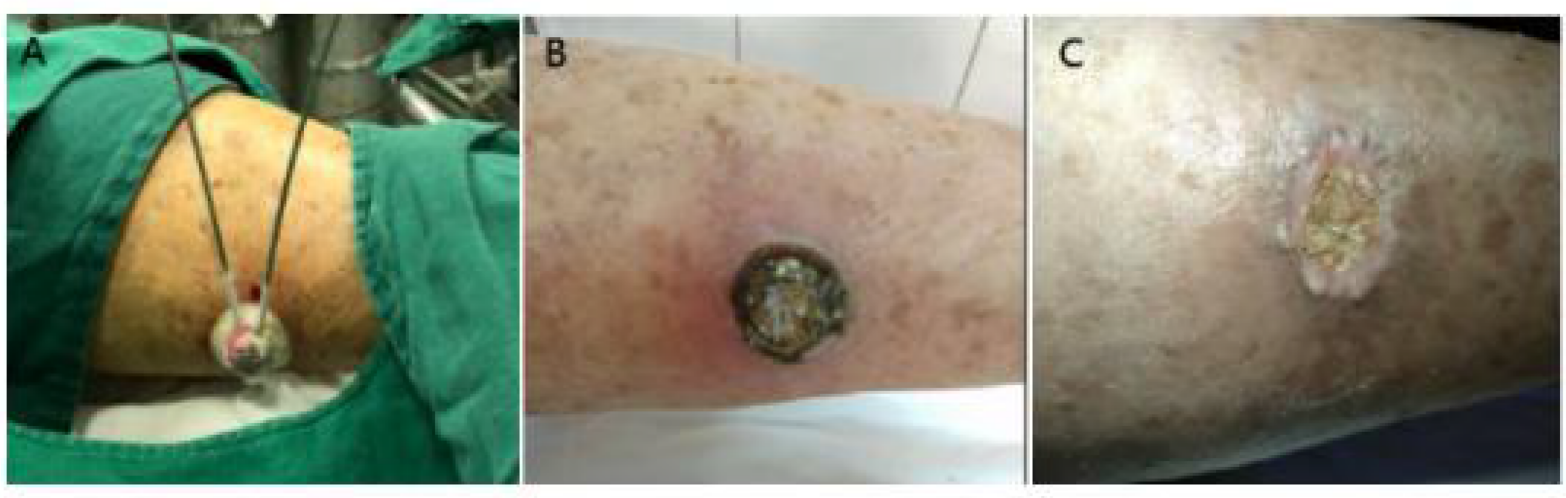
**(A)** Case 5 during cryotherapy. **(B)** Two weeks after cryotherapy. **(C)** Six weeks after cryotherapy and complete wound healing.

**Figure 3:**
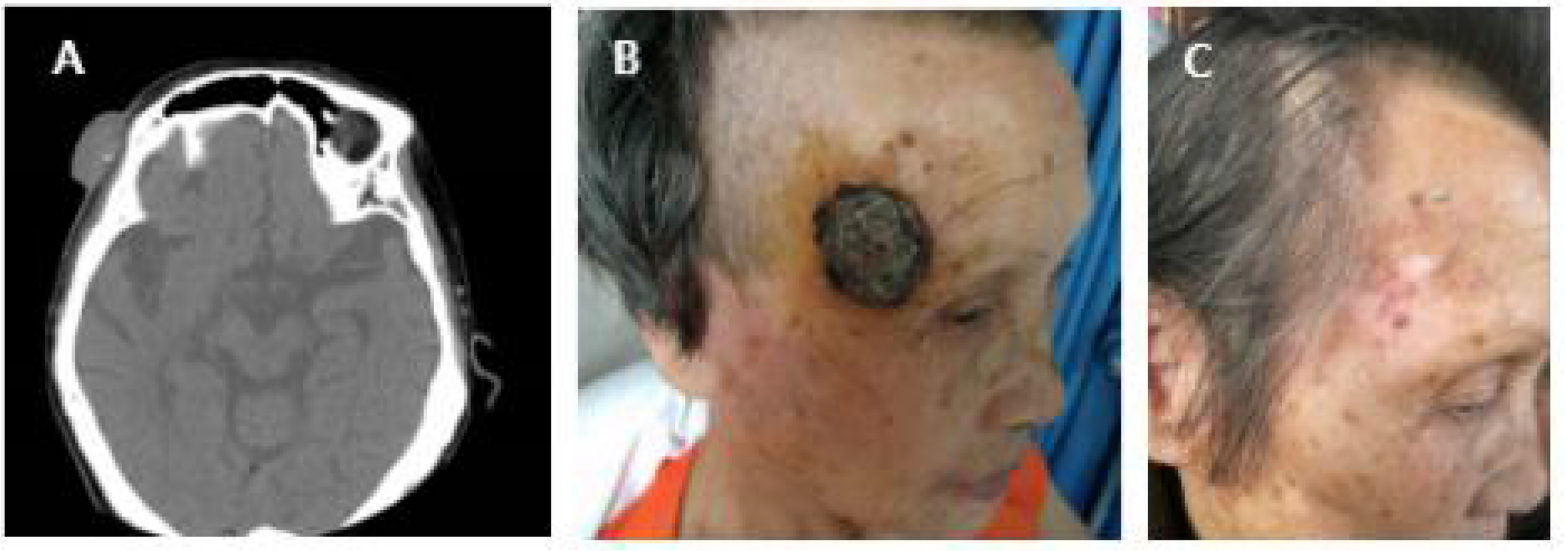
**(A)** Case 4 before cryotherapy. **(B)** One week after cryotherapy. **(C)** Three months after cryotherapy. The patient was followed up for 5 years without recurrence.

**Figure 4:**
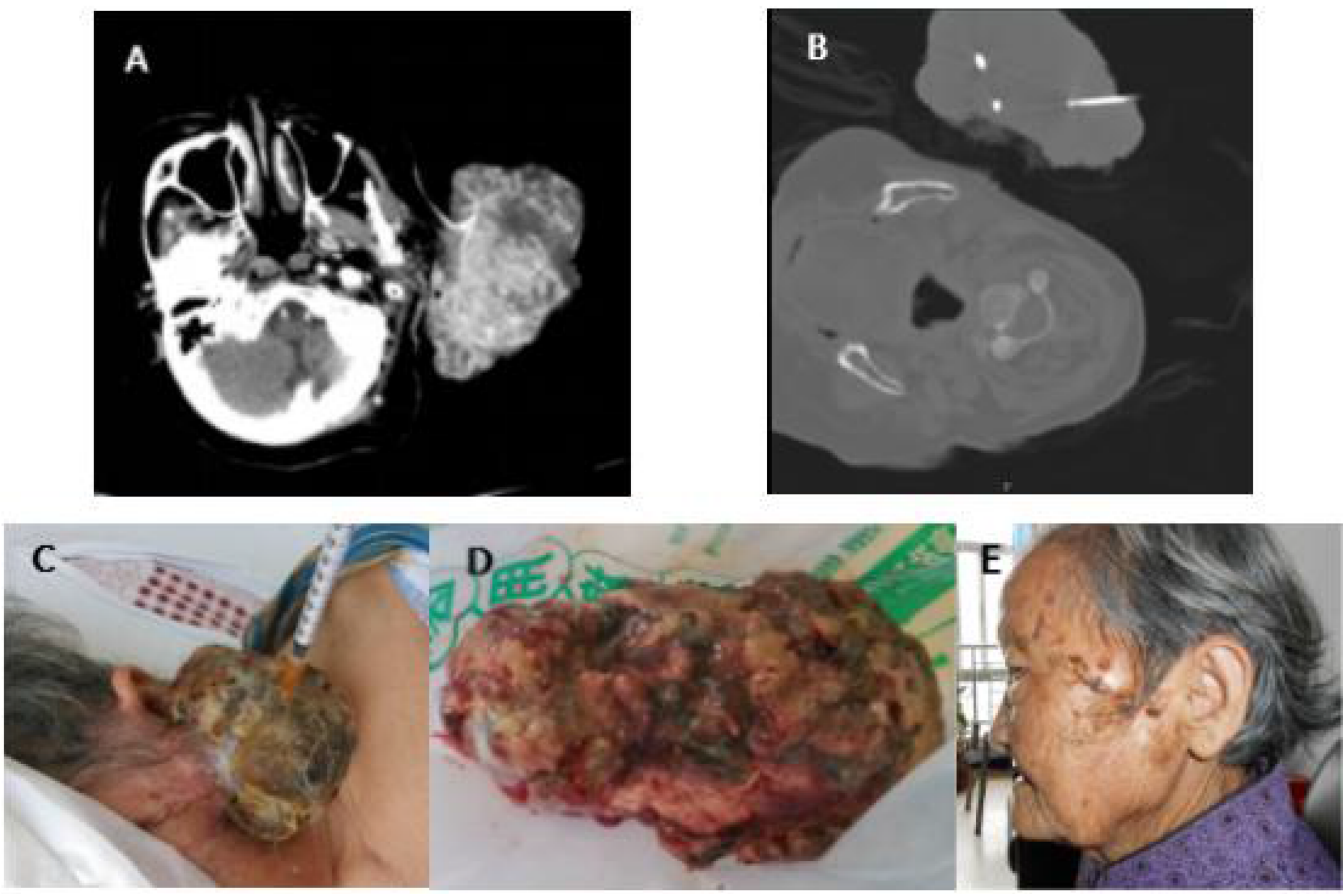
**(A)** Case 1 before cryotherapy. **(B)** During cryotherapy. **(C)** Two days after cryotherapy and tumor tissue necrosis. **(D)** Three days after cryotherapy, due to tumor necrosis, the patient developed infection and fever, and the necrotic tissue was finally removed by surgery. **(E)** The infection was controlled, and the wound of the cryoablation scab was healed (6 months after cryotherapy). The patient was followed up for 5 years without recurrence.

Each ablation session consisted of two freeze–thaw cycles. Complete ablation was achieved in all patients after a single ablation session. All patients experienced cryoablation, necrosis, scab, shedding, and healing (**Figures 2**, **3**). The whole process lasted 4–8 weeks. Case 1 was a patient with a tumor measuring 15.5 cm × 10.5 cm who developed postoperative infection and fever because of extensive necrosis after cryoablation. Surgery was performed to remove the necrotic tissue. The infection was eventually controlled, and the wound of cryoablation scab healed (**Figure 4**).

No severe complications occurred during cryotherapy. The major postoperative complications were pain and hemorrhage. Slight pain after ablation was reported by all patients, but the pain was tolerable and did not require medication. Three patients experienced slight hemorrhage at the cryoprobe puncture site after ablation, but it ceased after 3–5 min of compression with gauze.

All patients achieved satisfactory cosmetic results. No plastic surgery was performed on any patient. The quality of life of the patients improved significantly.

The follow-up study lasted for 5 years. During this period, two patients passed away due to unrelated diseases 3 years after the therapy. None of the 6 patients had a recurrence.

## Discussion

In this study, we reported satisfactory safety and clinical efficacy of CT-guided argon–helium cryoablation for elderly cSCC with medium- and large-sized tumors. Complete ablation and clinical full recovery were achieved after a single ablation session in all recruited patients, and the procedure-related complications were controllable. Our results suggested that the use of argon–helium cryoablation procedure was a promising alternative or complement to resection for the elderly cSCC patients with large-sized tumors and not suitable for conventional surgery.

Argon–helium cryoablation is a new surgical treatment for malignant tumors, especially for unresectable tumors. It is a minimally invasive treatment, and the tumor is destroyed by freeze–thaw cycles. The direct and deep freeze of the lesions causes protein denaturation and microvascular thrombosis. The subsequent thaw section causes the burst of the cells and eventually results in the death of tumor cells (13, 14). Furthermore, the repeated freeze–thaw cycle causes membrane disruption of the tumor cells, which promotes releasing of the hidden tumor-derived self-antigens into the circulation, stimulates antibody production, and activates the antitumor immunity of the body (15, 16). Various studies indicate the anti-neoplastic and immune-logical effects of argon–helium cryoablation (17, 18).

This was the first study reporting the effectiveness of argon–helium cryoablation in the clinical treatment of cSCC. Liquid nitrogen cryotherapy is one of the common cryotherapy methods for the treatment of cSCC. However, it is more widely used for malignant tumors with superficial location, small diameter (less than 2 cm), and low metastatic risk (4, 19, 20). In this study, the argon–helium cryoablation was used for the treatments of elderly cSCC patients with large tumor sizes. In the results, we found that all patients experienced tumor necrosis, scab, and natural peeling process after cryoablation. The full recovery period was 4–8 weeks. The most frequently reported adverse reactions were mild pain and slight hemorrhage that were controllable. All patients obtained good cosmetic effects. The follow-up study lasted for 5 years. During this period, 4 patients were alive and 2 patients passed away due to unrelated diseases. This study demonstrated good safety, high effectiveness, satisfactory cosmetic outcome, short recovery period, and convenient and fast surgery procedure of argon–helium cryoablation in the clinical treatment of cSCC in the elderly.

Argon–helium cryoablation has been widely used in the clinical treatment of liver cancer, lung cancer, kidney cancer, breast cancer, bone tumors, prostate cancer, and tumors of other organs (13, 21–27). Several reports demonstrated that argon–helium cryoablation produced clinical efficacy and long-term outcomes equivalent to surgery in patients with a single tumor up to 5 cm in diameter (28–30). Consistently, our results suggested satisfactory treatment outcomes for patients with cSCC following argon–helium cryoablation. No recurrence of the tumor was found in all the patients. Even if recurrence was to occur, cryoablation is easy to repeat.

In conclusion, as suggested by our results, argon–helium cryoablation may be a promising alternative therapy for elderly cSCC patients with large tumors. Elderly patients are often not suitable for standard surgical treatment, radiotherapy, or systemic therapy. Argon–helium cryoablation possessed the advantages of minimal invasion, mild adverse reactions, short hospital stay, rapid recovery, good cosmetic outcome, and less damage to the surrounding normal tissues. Despite the promising results, this study was limited by the small sample size. Further studies with larger clinical sample sizes are necessary to validate the clinical efficacy of argon–helium cryoablation in the treatment of cSCC.

## Data Availability Statement

The original contributions presented in the study are included in the article/supplementary material. Further inquiries can be directed to the corresponding author.

## Ethics Statement

The studies involving human participants were reviewed and approved by the medical ethics committee of Boluo County People’s Hospital. The patients/participants provided their written informed consent to participate in this study. Written informed consent was obtained from the individual(s) for the publication of any potentially identifiable images or data included in this article.

## Author Contributions

QH conceived and designed the study, performed the data collection and analysis, and wrote most of the manuscript. WX is the principal investigator of the study and helped write a portion of the manuscript and review it. All authors contributed to the article and approved the submitted version.

## Conflict of Interest

The authors declare that the research was conducted in the absence of any commercial or financial relationships that could be construed as a potential conflict of interest.

## Publisher’s Note

All claims expressed in this article are solely those of the authors and do not necessarily represent those of their affiliated organizations, or those of the publisher, the editors and the reviewers. Any product that may be evaluated in this article, or claim that may be made by its manufacturer, is not guaranteed or endorsed by the publisher.
